# Victim Sensitivity and Altruistic Behavior in School: Mediating Effects of Teacher Justice and Teacher-Student Relationship

**DOI:** 10.3389/fpsyg.2019.01077

**Published:** 2019-05-08

**Authors:** Shuyang Jiang, Ru-De Liu, Yi Ding, Tian Po Oei, Xinchen Fu, Wei Hong

**Affiliations:** ^1^ Beijing Key Laboratory of Applied Experimental Psychology, National Demonstration Center for Experimental Psychology Education, Faculty of Psychology, Beijing Normal University, Beijing, China; ^2^ Graduate School of Education, Fordham University, New York, NY, United States; ^3^ School of Psychology, University of Queensland, St Lucia, QLD, Australia; ^4^ Department of Psychology, James Cook University, Singapore, Singapore

**Keywords:** victim sensitivity, altruistic behavior, teacher justice, teacher-student relationship, adolescents

## Abstract

The current study aimed to explore how victim sensitivity influenced altruistic behaviors in school and to explore the mediating roles of teacher justice and teacher-student relationship. In 2018, we recruited 1,856 Chinese adolescents including 989 fourth graders (*M* = 10.35, SD = 0.56) and 867 eighth graders (*M* = 15.57, SD = 0.91), and the participation rate was 100%. Participations completed the self-report victim sensitivity scale, the teacher justice scale, the teacher-student relationship scale, and the altruistic behavior toward classmate scale. Structural equation modeling (SEM) indicated that victim sensitivity had a direct negative effect on altruistic behavior in school, but this relationship was mediated by teacher justice. There was also a mediated path between teacher justice and altruistic behavior by way of teacher-student relationship. These findings suggested possible mechanisms to explain the relationship between victim sensitivity and altruistic behavior and provided new directions for intervention.

## Introduction

Altruistic behavior refers to people share their own resources and energy with others without expecting any rewards (Batson, 1991, 2010; Kurzban et al., 2015). A growing number of papers on adolescents have documented that altruistic behavior may serve to enhance students’ school achievement, reduce school dropout, physical violence, bullying, and mental health problems (Kokko et al., 2006; Raskauskas et al., 2010; Haroz et al., 2013). Others have indicated that altruistic behavior is a robust predictor of children’s adjustment outcomes and psychosocial well-being (Flynn et al., 2015).

Given the important role of altruistic behavior in personal development, many researchers have sought to identify factors that could predict individuals’ altruistic behavior. However, few studies have explored the predictors of altruistic behavior in educational contexts, such as schools. In fact, schools are socializing venues that can nurture “character strengths” such as compassion, altruism, and social equity (Yates and Youniss, 1996). Additionally, individuals who perform altruistic behaviors can not only reduce peer rejection and increase quality of peer relationships (Carlo et al., 2010; Wang et al., 2015b) but can also cause pro-social values and attitudes to accrue in observers, motivating the observers to perform altruistic behaviors (Hardy and van Vugt, 2006; Fehrler and Przepiorka, 2013; Luengo Kanacri et al., 2017), which further foster positive school climates and prevent school violence. Thus, the current study will examine the predictors and the underlying mechanism of altruistic behavior within a school context.

### Victim Sensitivity and Altruistic Behavior

Victim sensitivity has been proposed to have a link with altruistic behavior (Baumert et al., 2012). Victim sensitivity refers to an individual’s tolerance of unjust treatment toward themselves (Schmitt et al., 2005, 2010). Research on personality-congruent information processing has indicated that personality functions as a guide in directing information processing, shaping emotions, and behavioral tendencies in various domains (Rusting, 1998; Mor and Inbar, 2009). For example, studies have demonstrated that individual characteristics (e.g., age, narcissism, empathy, and personality disorder) can significantly affect one’s emotion recognition process (Daros et al., 2013; Konrath et al., 2014; Guarnera et al., 2018). Similarly, victim sensitivity has been linked to biased processing of justice-related information, which leads to fewer altruistic behaviors and more destructive behaviors (Fetchenhauer and Huang, 2004; Gollwitzer and Rothmund, 2009). Specifically, people with high victim sensitivity tend to view the world through a suspicious mindset, and they are always alert to others who might exploit them (Gollwitzer et al., 2005). It is suggested that victim sensitivity may serve as a motivating force urging individuals to find ways to protect their own interests. Moreover, empirical studies have testified that high victim sensitivity would lead to reduce willingness to engage in solidary behaviors and increase immoral thoughts and actions (Gollwitzer and Rothmund, 2009; Maltese et al., 2016). Accordingly, it is suggested that students with high victim sensitivity are uneasy about being exploited by others and thus may perform fewer altruistic behaviors.

While victim sensitivity may decrease students’ tendency to altruistic behavior, the psychological mechanism linking these two variables remains unclear. Thus, the present study aims to elucidate the underlying mechanism of the relationship between victim sensitivity and altruistic behavior.

### Teacher Justice and Teacher-Student Relationship as Mediators

One possible explanation for the negative association between victim sensitivity and altruistic behavior concerns teacher justice. Teacher justice is one of the most typical school justice experiences (Peter and Dalbert, 2010) because teachers not only take charge of students’ academic evaluation but also possess authority on other privileges and punishments (Jiang et al., 2018). Students form their perception of teacher justice through interactions with teachers during educational activities, which largely depend on individuals’ perception, processing, and evaluation of relevant information (Dalbert, 2001; Peter et al., 2013). Following the Sensitivity to Mean Intentions (SeMI) model, victim-sensitive individuals have a more suspicious mindset, which guides information processing and behavior orientation (Gollwitzer and Rothmund, 2009). To be specific, victim-sensitive individuals could consider even subtle or meaningless untrustworthiness cues to reflect mean intentions. Such individuals are motivated to avoid being exploited and thus perform fewer altruistic, collaborative, and cooperative behaviors (Gollwitzer and Rothmund, 2009; Gollwitzer et al., 2013). This theoretical model has been demonstrated in several studies (Traut-Mattausch et al., 2011; Baumert et al., 2012; Maltese et al., 2016). For example, in a workplace environment, researchers found that victim-sensitive individuals tended to perceive more unfair treatment by their employers (Schmitt and Dörfel, 1999). It is indicated that for students in educational contexts, victim sensitivity may serve to change the meaning of ambiguous teacher behavior, leading to lower teacher justice perceptions. Furthermore, students’ experience of teacher injustice was negatively related to their group identification (Jiang et al., 2018) and positively related to problem behaviors, such as bullying, cheating, and delinquent behavior (Donat et al., 2012, 2014), which further reduced students’ altruistic behaviors. Based on these findings, it can be argued that teacher justice is an important mediating factor for the association between victim sensitivity and altruistic behavior in school.

Another important factor that can affect altruistic behavior is teacher-student relationship. According to social disorganization theory (Shaw and McKay, 1942), within a community, breakdown of institutions (e.g., school, class) can produce deviant behaviors, which can not only limit the capacity to supervise members’ behavior but can also work against fostering positive and cooperative relationships (Wang et al., 2015a). Moreover, social control theory (Hirschi, 1969) suggests that adolescents are more likely to engage in delinquent behavior when they fail to bond to society (in the form, e.g., of adherence to social rules or relationships with important people). These theories both emphasize the importance of social norms and relationships to important people to one’s behavior in the community (Wang et al., 2015a). It follows that, for students, teacher justice and teacher-student relationship may both be critical for students’ altruistic behavior in school. Teacher justice is the result of teachers implementing school norms to avoid unexpected behaviors, such as rule breaking, aggression, and bullying (Donat et al., 2012; Molinari et al., 2013). When students think their teachers treat them unjustly, they may consider themselves to be excluded and undervalued (Jiang et al., 2018), which will in turn disrupt the formation of secure relationships with their teachers. For instance, Molinari et al. (2013) found that students who experienced lower teacher justice were more likely to report that their teachers created hostile relations rather than cooperative ones. Moreover, good teacher-student relationships have been recognized as protective for positive social interactions among students, such as peer acceptance and school adjustment (Longobardi et al., 2016), which promote students’ positive attitude and sense of belonging at school (Hughes, 2011). Thus, students become more active participants in school affairs and are ultimately more willing to engage in altruistic behaviors. Thus, we assume that victim sensitivity may reduce students’ perceptions of teacher justice, damage teacher-student relationships, and make students less likely to perform altruistic behaviors.

### The Present Study

Prior studies have shown that victim sensitivity can be an antecedent variable that affects altruistic behavior, and similar results have been observed in social decision-making settings (Maltese et al., 2016). The present study extended such findings to the Chinese educational context. Altruism is regarded as an important quality in Chinese traditional culture and has been widely valued in schools’ education (Ding and Song, 2017). Thus, we focused on both primary school (fourth grade) and middle school (eighth grade) students. It was assumed that victim sensitivity would reduce altruistic behaviors.

**Figure 1:**
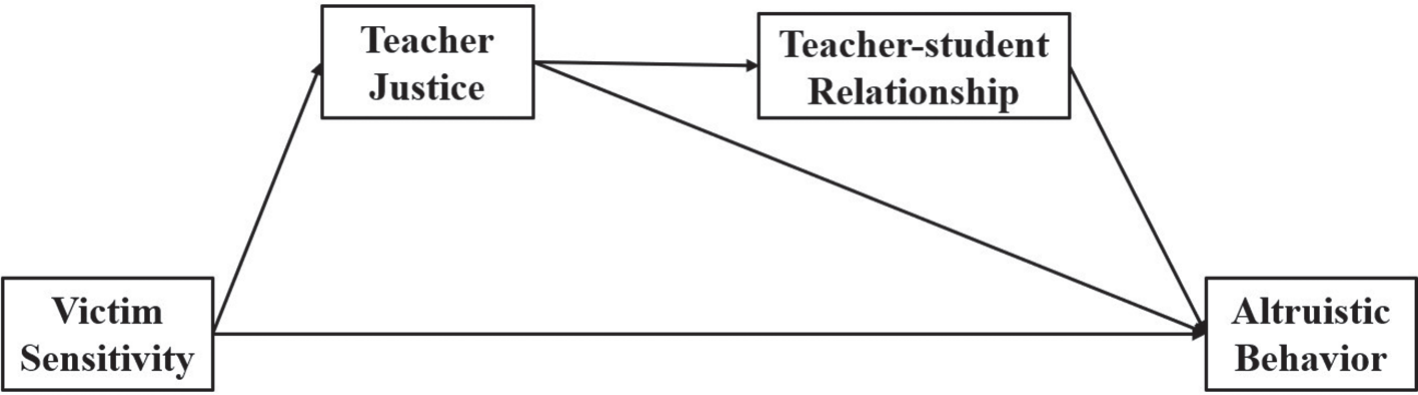
The hypothesized model.

In addition, we also tested the mediating roles of perception of teacher justice and teacher-student relationships in the relationship between victim sensitivity and altruistic behaviors. We hypothesized that perception of teacher justice and perceived teacher-student relationships act as mediators for the relationship between victim sensitivity and altruistic behaviors in school settings. The model to be tested is presented in Figure 1.

## Materials and Methods

### Participants and Procedure

The data were collected as a part of a national educational research project among Chinese adolescents. In the present study, we first contacted the local educational agencies and the principals of the middle schools in a city of Eastern China, and we informed them the purpose of our study. We received approval to recruit all students in Grades 4 and 8 based on students’ voluntary participation. All students who were present on the day of assessment were able to participate in the study. In total, 989 fourth graders (546 boys, 443 girls, ages 10–12 years, *M* = 10.35, SD = 0.56) and 867 eighth graders (444 boys, 423 girls, ages 13–15 years, *M* = 15.57, SD = 0.91) participated in the study.

The current study was approved by the Research Ethics Committee of a major research university in China and by the principals of the participating schools. Before the formal investigation was conducted, all students and their parents were informed of the research purpose and the voluntary nature of participation. Written consent forms were distributed to the students’ parents. Parents were asked to sign the consent forms for their children to participate in our study. Data collection was completed during class time with the help of school teachers. Participants were assured that all personal information and their responses would be kept confidential and would be used for research purposes only.

### Measures

#### Victim Sensitivity

Victim sensitivity was measured using the Victim Sensitivity subscale of the Justice Sensitivity Inventory. The original questionnaire was developed by Schmitt et al. (2010), and we adapted this scale into Chinese version. The subscale consisted of 10 items, and a sample item included, “It bothers me when others receive something that ought to be mine.” Participants responded on a Likert-type scale ranging from 0 (not true at all) to 5 (very true). The subscale had good internal reliability in prior research among Chinese sample (*α* = 0.91; Liu et al., 2016) as well as in the present study (*α* = 0.88).

#### Teacher Justice

The teacher justice toward oneself (TJ-self) subscale from teacher justice questionnaire (Gorard, 2012) was used to measure students’ experience about teacher justice. The subscale consisted of seven items (sample item: “I was always treated fairly by my teachers”). Each item was rated using a 5-point Likert-type scale ranging from 1 (not true at all) to 5 (very true of me). The items were found to be reliable and valid in prior research (Jiang et al., 2018). In this study, the scale had satisfactory internal consistency (*α* = 0.84).

#### Teacher-Student Relationship

We measured teacher-student relationship using the Inclusion of Other in the Self (IOS) scale (Aron et al., 1992). The IOS is a single-item pictorial measure and consists of seven Venn-like diagrams depicting different degrees of overlap between two circles. Participants were instructed to choose the picture that best describes their relationship with their teachers. This scale has demonstrated adequate psychometric properties and good predictive validity (Agnew et al., 2004).

#### Altruistic Behavior

A four-item questionnaire was used to measure students’ altruistic behavior toward classmates. The original scale was the altruism toward colleague questionnaire developed by Farh et al. (1997). Considering their applicability to Chinese adolescents, we adapted the items by changing the word “colleagues” into “classmates” and slightly modified the expression (sample item: “Willing to assist new classmates to adjust to the school environment.”). Each item was measured on a 7-point Likert-type scale. The internal reliability of the scale in this study was adequate (*α* = 0.81).

### Data Analysis

We first evaluated the pattern of missing data. The results showed that 0.9% of data were missing, and the missing rates on all cases were less than 22.7% (much lower than 50%), which indicated that their data could be retained. Additionally, we analyzed the type of missing data using Little’s Missing Completely at Random (MCAR) test in SPSS 23.0 software. The result revealed that the patterns of missingness did not meet the strict criteria of MCAR. Therefore, robust maximum likelihood estimation (MLR) was selected as the method of imputation of the missing data (Wang and Wang, 2012) in the following structural equation model.

We conducted descriptive analyses for each measure and calculated Pearson’s correlations between the main measures using SPSS 23.0 software. To examine the mediating effects of teacher justice and teacher-student relationship on the relationship between victim sensitivity and altruistic behavior, we performed structural equation modeling (SEM) using Mplus 7.0 software. Following the work of Hu and Bentler (1999), we adopted the following indices to evaluate the model fit: Comparative Fit Index (CFI), Tucker-Lewis Index (TLI), the Standardized Root Mean Square Residual (SRMR), and the Root Mean Square Error of Approximation (RMSEA). In addition, a two-step procedure was conducted to examine the mediating roles of teacher justice and teacher-student relationship in the relation between victim sensitivity and altruistic behavior. First, after controlling for grade and gender, we built a direct effect model to assess the direct effect of victim sensitivity on altruistic behavior. Second, we added teacher justice and teacher-student relationship as mediators. We further conducted bootstrapping analysis to establish confidence intervals (CIs) for multiple indirect effects.

## Results

### Descriptive Statistics and Correlation Analysis

Descriptive statistics and Pearson’s correlations for the main variables are presented in Table 1. We found that gender was positively associated with victim sensitivity and negatively associated with teacher justice, teacher-student relationship, and altruistic behavior. Furthermore, grade was found to have a significant positive relation with altruistic behavior, but it was not significantly correlated to other variables. Except for gender and grade, victim sensitivity was negatively correlated with teacher justice, teacher-student relationship, and altruistic behavior. Moreover, teacher justice, teacher-student relationship, and altruistic behavior were positively correlated with each other.

**Table 1:** Means, standard deviations, and correlations among main variables.

	*M* ± SD	1	2	3	4	5	6
1. Grade	–	–					
2. Gender	–	–	–				
3. Victim sensitivity	3.38 ± 0.55	0.38^***^	0.00	–			
4. Teacher justice	2.96 ± 0.67	−0.49^***^	0.03	−0.27^***^	–		
5. Teacher-student relationship	2.61 ± 0.74	−0.19^***^	−0.01	−0.16^***^	0.37^***^	–	
6. Altruistic behavior	3.25 ± 0.58	−0.17^***^	0.08^**^	−0.13^***^	0.40^***^	0.30^***^	–

** p < 0.01;

*** p < 0.001.

### Examination of Mediating Effects

SEM was used to analyze the mediation effect. First, we examined the direct effect of victim sensitivity on altruistic behavior. The direct model showed a good fit to the data (χ^2^/df = 7.48, CFI = 0.943, TLI = 0.932, RMSEA = 0.059, SRMR = 0.045). The results revealed that victim sensitivity had a negative and significant effect on altruistic behavior (*β* = −0.12, *p* < 0.001).

**Figure 2:**
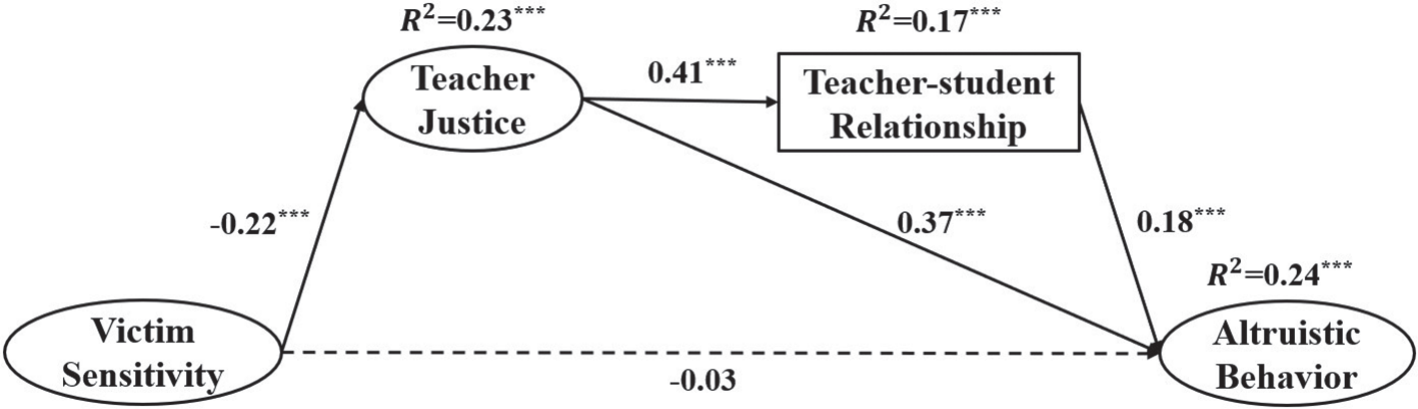
The mediating effects model after controlling for grade and gender. Factor loading is standardized, ^***^ *p* < 0.001.

Afterward, to test our hypothesis, we added teacher justice and teacher-student relationship as mediators between victim sensitivity and altruistic behavior. The multiple indirect effect model also showed a good fit to the data (χ^2^/df = 4.90, CFI = 0.945, TLI = 0.939, RMSEA = 0.046, SRMR = 0.043), see Figure 2.

Furthermore, we used bootstrapping to calculate the 95% confidence intervals (2000 resamples) to assess the significance of the indirect effects (see Table 2). As shown in Table 2, the indirect effect of victim sensitivity on altruistic behavior through teacher justice was significant, as well as the chain mediating effect of teacher justice and teacher-student relationship.

**Table 2:** Bootstrap analyses of mediating effects.

Model pathways	Estimated effect	95% CI
Direct effect		
Victim sensitivity→ Altruistic behavior	−0.03	−0.08, 0.02
Indirect effect		
Victim sensitivity→ Teacher justice→ Altruistic behavior	−0.081	−0.10, −0.05
Victim sensitivity→ Teacher justice→ Teacher-student relationship → Altruistic behavior	−0.016	−0.02, −0.01
Total indirect effects	−0.098	−0.12, −0.06

## Discussion

The present study aimed to investigate the relationship between victim sensitivity and altruistic behavior in Chinese students. The result of SEM showed that victim sensitivity has a significant effect on students’ altruistic behavior, in line with our hypothesis and replicating the findings of previous studies (Maltese et al., 2016). Schmitt et al. (2010) proposed that individuals of high victim sensitivity have a suspicious mindset that promotes selfish and even antisocial behavior. We further examined the underlying mediating effects of teacher justice and teacher-student relationship.

The result of SEM and bootstrap analysis indicated that teacher justice mediates the influence of victim sensitivity on altruistic behavior. Students with high victim sensitivity tend to expect unfair treatment (Schmitt et al., 2010). For example, they may magnify the untrustworthiness cues that their teachers display in daily interactions, including through class arrangement, duty distribution, and performance assessment. During such activities, such students are easily threatened by any possible unjust cues, and hence, they are more likely to negatively assess teacher justice. Low justice perception may further cause students to feel alienation from their groups through reduced class identification, school belonging, and social trust (Resh and Sabbagh, 2014; Jiang et al., 2018), which consequently decrease students’ willingness to engage in altruistic behaviors in school. This phenomenon is illustrated by the Chinese proverb, “If the upper beam is not straight, the lower ones will go aslant.” When students think teachers treat them unjustly, their belief in teachers’ authority and credit will be weakened, making it more difficult for them to internalize teachers’ values (e.g., helping and caring) and to behave as teachers’ expect (Wang et al., 2015a).

Additionally, the results revealed that teacher justice exerted a positive effect on altruistic behavior through teacher-student relationship, in accordance with our hypothesis and existing theories (Shaw and McKay, 1942; Hirschi, 1969). Students’ evaluation of teacher justice depends on whether teachers’ allocation of rewards or punishments aligns with students’ personal constructed perceptions of deservedness (Resh and Sabbagh, 2014). According to the Group-Value-Theory (Lind and Tyler, 1988), students’ experiences of teacher injustice not only serve as a signal that the students are considered undervalued members of the group, which makes them feel ignored and rejected (Donat et al., 2018), but also trigger strong behavioral responses such as anger, rule breaking, and aggression (Donat et al., 2012). Considering the dynamic nature of student-teacher relationship, it is extremely stressful for teachers to deal with students’ misbehaviors, negative emotions, and failures in the teaching process (Di Chiacchio et al., 2016; Fiorilli et al., 2017). To manage such undesired behaviors, teachers experience high level of stress, lack of sense of efficacy, and burnout (Emmer and Stough, 2001; Aloe et al., 2014), and they use more punitive classroom practices (Bibou-Nakou et al., 1999; Jennings and Greenberg, 2009). Such interactions intensify the conflict between teachers and students and result in unhealthy teacher-student relationships (Aloe et al., 2014; Fiorilli et al., 2017). Unhealthy teacher-student relationships have an adverse effect on students’ development of social skills (Pianta et al., 2008) and inhibit positive and prosocial attitudes in the classroom (Longobardi et al., 2016), resulting in less altruistic behavior (Jennings and Greenberg, 2009; Eisenberg et al., 2015).

More importantly, the results indicated that the relationship between victim sensitivity and students’ altruistic behavior was completely explained by teacher justice and teacher-student relationships. Specifically, victim sensitivity impedes students’ altruistic behavior, and this relation can be mediated in two ways: (1) victim sensitivity lowers students’ perception of teacher justice and (2) a negative teacher-student relationship created by perceived teacher injustice. These results highlight that teacher justice and teacher-student relationship are both powerful bonds that strength the psychological contract between students and their groups (Wang et al., 2015a; Jiang et al., 2018). This is especially noteworthy in the context of China because Chinese students usually stay in a stable group (a class) for an entire school cycle (e.g., primary school, for 6 years; middle school, for 3 years), and the same class teacher usually teaches the same class for the same period (Chang et al., 2004). In such a setting, the interactions and relationships between teachers and students are formed early on and cannot easily be changed, making the relationships even more crucial for students’ altruistic behavior in school.

To our knowledge, this is the first study to investigate the underlying mechanism by which victim sensitivity influences altruistic behaviors in the context of school based on large sample of primary and middle school students. The results are consistent with the basic assumptions of personality-congruent information processing studies (Rusting, 1998; Thomas et al., 2011) and provide a more detailed picture of how students’ justice perception, relationship development, and behavioral choices could be influenced by victim sensitivity. It is worth noting that victim sensitivity can lead to patterns of negative social interaction, which is strongly associated with psychological and behavioral problems (e.g., depression and internalizing problems; Jellesma et al., 2015; Luo et al., 2017), as well as school bullying and maladjustment (Sarkova et al., 2014; Longobardi et al., 2016). In this regard, victim sensitivity seems to be a potential risk factor for adolescents’ psychological well-being. Furthermore, the findings also provide possible guidelines for teachers to improve students’ school experience by helping to promote altruistic behaviors. For instance, teachers should be aware of the significance of teacher justice and teacher-student relationship in establishing the psychological bond between students and their groups. For example, teachers can foster an equal and harmonious atmosphere by being just in grading, providing explanations for reward and punishment, and encouraging students’ participation in group decision making (Peter and Dalbert, 2010). In this way, students will feel more secure and related to their environment and subsequently perform more altruistic behaviors.

The present study has several limitations. First, the study mainly focused on students’ appraisal of teacher-student relationships. Future research should adopt a multi-assessment approach to replicate our results and explore whether students with high victim sensitivity underestimate the quality of their relationships with teachers. Second, our findings were generated from a cross-sectional research design, and we relied on theoretical assumptions and empirical evidence to construct our hypotheses and to interpret the predictive roles of variables in SEM. Further longitudinal research is needed to determine the causality of the model and the stability of this relationships across time. Finally, justice sensitivity may have distinctive cultural characteristics (Schmitt et al., 2010). The measurement used in the current study was designed for Western contexts. Thus, an assessment instrument for collectivistic cultures must be developed.

## Conclusion

This study enriches our understanding of the relationships between individuals’ dispositional victim sensitivity and altruistic behaviors in the context of school. Our findings showed that the relation between victim sensitivity and altruistic behaviors is mediated by teacher justice and teacher-student relationships. These results help us to understand the possible mechanisms by which victim sensitivity decreases altruistic behaviors in school.

## Ethics Statement

This study was approved by the Research Ethics Committee of Beijing Normal University. All subjects gave written informed consent in accordance with the Declaration of Helsinki.

## Author Contributions

All the coauthors are participants in the data collection, data analysis, writing, and revising the manuscript.

### Conflict of Interest Statement

The authors declare that the research was conducted in the absence of any commercial or financial relationships that could be construed as a potential conflict of interest.
